# Vitamin B12 deficiency neuropathy; a rare diagnosis in young adults: a case report

**DOI:** 10.1186/s13104-017-2393-3

**Published:** 2017-01-28

**Authors:** Cyril Jabea Ekabe, Jules Kehbila, Martin Hongieh Abanda, Benjamin Momo Kadia, Carlson-Babila Sama, Gottlieb Lobe Monekosso

**Affiliations:** 1 Grace Community Health and Development Association, P. O. Box 15, Kumba, Southwest Region Cameroon; 2 Wum District Hospital, Wum, Northwest Region Cameroon; 3 Non-Communicable Diseases Unit, Clinical Research Education, Networking & Consultancy (CRENC), Douala, Cameroon; 4 Presbyterian General Hospital Acha-Tugi, Acha, Northwest Region Cameroon; 5 Galactic Corps Research Group (GCRG), Buea, Southwest Region Cameroon; 6 Bambalang Sub-divisional Hospital, Bambalang, Northwest Region Cameroon; 7 Department of Medicine, Faculty of Health Sciences, University of Buea and Global Health Dialogue Foundation, Buea, Cameroon

**Keywords:** Vitamin B12 deficiency, Neuropathy, Young female, Resource–limited setting, Cameroon

## Abstract

**Background:**

Vitamin B12 deficiency is a metabolic disorder with many causes. It often presents with megaloblastic anaemia and neurological disorders which entail prompt treatment. The diagnosis of Vitamin B12 deficiency is challenging in resource limited-settings due to limited access to diagnostic tools and unfamiliarity with the disease, owing to its rarity especially in young people.

**Case presentation:**

A 28 year old female Cameroonian presented with progressive burning painful sensations on the upper trunk, paraesthesia and numbness of the upper and lower limbs for a period of 5 years. Before presenting to us, she had consulted in numerous health institutions for which she had been treated for diverse pathologies with no relieve of symptoms. After clinical and laboratory evaluation, a diagnosis of vitamin B12 deficiency-associated neuropathy was made. She was placed on oral vitamin B12 supplements at 2 mg daily for 3 months. Follow up was marked by good clinical recovery after 1 month of therapy.

**Conclusion:**

Vitamin B12 deficiency neuropathy is a rare debilitating disease that affects mostly the elderly. However; young adults with neuropathic symptoms warrant a high index of suspicion. Peripheral blood smears and complete blood counts are sufficiently diagnostic in resource-limited settings.

## Background

Vitamin B12 (VB12) is an essential water soluble vitamin important in cellular metabolism and maintenance of the integrity of the nervous system [1]. It is important in the synthesis of DNA and thus cell division [2]. Deficiency in vitamin B12 is associated with impaired erythropoiesis and nervous system demyelination, which account for most of its clinical manifestations [3, 4]. Globally, the most common aetiology of vitamin B12 deficiency is lack of intrinsic factor in patients with pernicious anaemia; a common finding in the elderly [4]. Other aetiologies include dietary deficiency and mal-absorption [5].

Clinically, VB12 deficiency presents mainly with neurological and psychiatric manifestations [5]. Neurological impairment usually presents as paraesthesia, numbness and ataxia [5, 6]. Laboratory investigations play an important role in the diagnosis and determination of the aetiologies of this deficiency. Laboratory investigations vital in diagnosing VB12 deficiency include mean corpuscular volume (MCV), peripheral blood smear (ovalomacrocytosis and hypersegmented neutrophils), cyanocobalamin levels, serum methylmalonic acid and homocysteine [7]. Therapeutic trials have also been used for the diagnosis of VB12 deficiency [7]. Early diagnosis of VB12 deficiency is pivotal for timely management and in preventing long term complications which could account for significant morbidity. We herein report a rare case of VB 12 deficiency in a young patient from a resource-poor setting.

## Case presentation

A 28 year-old sub-Saharan female presented with insidious onset of painful burning sensations on the upper trunk, symmetrical numbness and tingling sensation on the hands and feet, progressing over a 5 year-period. These symptoms began as intermittent burning sensations on the scapulae and pectoral regions of the upper trunk, which progressed to numbness and paraesthesia of the upper and lower limbs respectively. These were associated with mild weakness of the hands and feet, insomnia, irritability and constipation. There were no urinary symptoms, paralysis, gait disturbances, tremors, jaundice, limb swelling, changes in skin colour, or delusion. She had no known history of Human Immunodeficiency Virus (HIV) infection, diabetes, thyroid disease, syphilis, liver disease, renal disease, diphtheria, spinal cord injury or exposure to heavy metals. She is a laboratory technician and unmarried. She had been on omeprazole for 4 years for recurrent dyspepsia. She neither smokes cigarette nor consumes alcohol. She consumes meat regularly on weekly bases. A review of her previous consultations and laboratory results showed the following results; Aspartate amino transferase: 15 U/I, Alanine amino transferase: 22 U/I, Haemoglobin: 11 g/dl, HIV test: negative, fasting blood sugar: 97 g/dl. Spinal x-ray done 1 month prior to consultation was unremarkable. She had taken multivitamins, calcium and magnesium tablets, tramadol, prednisolone, and benzathin penicillin since the onset of illness. Despite these medications, there was no relieve in her symptoms.

On physical examination, her vital signs were stable. Her Conjunctivae were mildly pale, sclerae anicteric, and tonsils were normal. There were no skin changes, cervical adenopathy, or thyroid mass. Cardio-respiratory exam was normal. There was no hepato-splenomegaly or signs of liver disease. On examination of the neurological system, pupillary size and reflexes were normal. Cranial nerves were normal. Muscle tone and power were normal on both upper and lower limbs. Sensation to touch, pain and temperature were normal. There was impaired proprioception in both upper limbs. Tendon and cutaneous reflexes were normal. Lhermitte’s sign was absent. Based on her presentation we suspected a peripheral neuropathy with HIV peripheral neuropathy, neurosyphilis, and vitamin B12 deficiency as differentials. Blood tests showed Hemoglobin: 10.5 g/dl, Red blood cell count: 3 million/mcl, reticulocyte count: 0.3%, Mean Corpuscular Volume (MCV):110 fl, WBC: 8500 cells/μl, HIV test: negative, *Treponema pallidum* hematoglutinin assay (TPHA): negative, erythrocyte sedimentation rate (ESR): 10 mm/h, and peripheral blood smear analysis: ovalo-macrocytosis, and hyper-segmented neutophils. Stool exam and urinalysis were normal (Table 1). Serum cyanocobalamin levels were not assessed because of lack of diagnostic tools. A diagnosis of vitamin B12 deficiency related peripheral neuropathy was made based on her symptoms, ovalo-macrocytosis and hyper-segmented neutrophils on peripheral blood smear. She was treated with oral vitamin B12 tablets, at doses of 2 mg daily for 3 months. 1 month of therapy was marked with improvement in neurological symptoms and a follow up MCV of 97 fl, red blood cell count of 4.1 million/µl, and reticulocyte count of 0.95%.

**Table 1:** Laboratory results of a patient with Vitamin B12 deficiency neuropathy in a resource-limited setting of Cameroon

Laboratory test done	Results before treatment	Results after 1 month of treatment	Reference ranges
Hematological test			
RBC count	3 million cells/µl	4.1 million/µl	4–5.4 million/µl
Reticulocyte count (%)	0.3%	0.95%	0.5–1.5%
Hemoglobin (g/dl)	10.5 g/dl	12 g/dl	11–15 g/dl
MCV (fl)	110 fl	97 fl	80–95 fl
MCH (pg)	28 pg	30 pg	25–37 pg
Peripheral blood smear	Ovalomacrocytosis and hyper-segmented neutrophils (15%)	Ovalomacrocytosis and few hyper-segmented neutrophils (5%)	
WBC count	8500 cells/µl		4000–10,000 cells/µl
Platelet count	250000 cells/µl		150,000–450,000 cells/µl
Serological test			
TPHA	Negative		
HIV	Negative		
Stool examination	No ova or larva of parasites		
Urinalysis	Trace proteins		
Fasting blood sugar	89 g/dl		70–110 g/dl
Erythrocyte sedimentation rate (ESR)	10 mm/hr		

## Discussion and conclusion

Vitamin B12 is an essential water soluble vitamin which is important in cellular metabolism and maintenance of the integrity of the nervous system [1]. VB12 deficiency has numerous aetiologies which include; lack of intrinsic factor, nutritional deficiency, malabsorption and competition for VB12 [1, 4, 5].

The main neurological symptoms include: paraesthesia [8–12], ataxia [9–12], and limb weakness [9, 11]. The most prevalent psychiatric symptoms associated with B12 deficiency include, delusions [12, 13], irritability [13, 14], and decreased interest [15, 16]. Other manifestations included depression [12, 16] and sleep disturbances [12, 16]. These neurological symptoms are associated with other common illnesses like HIV infection, diabetes, syphilis, alcoholism, and some medications, thus posing a diagnostic challenge [17].

Basic laboratory investigations like full blood count (FBC) and peripheral blood smear analysis are vital in the diagnosis of this pathology [7] as evidenced by our case. Ovalo-macrocytosis and hyper-segmented neutrophils are important findings in VB12 deficiency [18], especially in settings were serum cyanocobalamin, methylmalonic and homocystein levels cannot be assessed. However, studies have designated that serum levels of VB12 can be unreliable for the assessment of VB12 deficiency [19]. Serum Transcobalamin II and methylmalonic acid levels are at present considered the most specific laboratory indices [19]. Nonetheless, Hyper-segmented neutrophils is reported to have a sensitivity of 98% [20], compared to serum cyanocobalamin with a sensitivity of 90–95% [21]. Thus, making peripheral blood smear analysis a cost effective tool in the diagnosis of VB12 deficiency, especially in settings like ours.

Anti-intrinsic factor and anti-parietal cell antibodies are important in diagnosing pernicious anaemia; a major aetiology of VB12 deficiency [7]. Conversely, mal-absorption from probable pernicious anaemia or chronic omeprazole use was the most likely aetiologies considered in this case. The response to oral VB12 supplement therefore pointed to chronic omeprazole use because intrinsic factor is needed for oral absorption.

The various methods of VB12 replacement therapy includes parenteral and oral [1]. Our patient was managed with oral VB12 tablets due to unavailability of the parenteral forms. It is known that VB12 is absorbed actively through its association with intrinsic factor. However, 1–2% of VB12 absorption occurs passively [22], thus oral replacement therapy can be as effective as parenteral therapy provided they are administered at high doses (2 mg daily) [23].

Therapeutic trials have also played vital roles in confirming VB12 deficiency, especially with follow up increases in MCV, RBC and Reticulocyte count [7, 24]. Follow up analysis of our case showed normalization of MCV, RBC and reticulocyte counts, which are important indices to assess therapeutic diagnosis and response in patients with VB12 deficiency.

This case emphasizes the importance of increased index of suspicion of Vitamin B12 deficiency in patients presenting with peripheral neuropathic symptoms. It also underscores the invaluable role of basic investigations like peripheral blood smear and the vitality of therapeutic trials which are pivotal to the timely detection and management of vitamin B12 associated neurological disease in resource-limited settings. Although this pathology is common in the old, young patients should be considered.

## Abbreviations

AIDS: acquired immune deficiency syndrome
DNA: deoxyribonucleic acid
HIV: human immunodeficiency virus
MCH: mean corpuscular haemoglobin
MCV: mean corpuscular volume
RBC: red blood cells
TPHA: treponema pallidum haematoglutinin assay
VB12: vitamin B12

## References

[1] Rusher DR, Pawlak R (2013). A review of 89 published case studies of vitamin B12 deficiency. J Hum Nutr Food Sci..

[2] Hvas AM, Nexo E (2006). Diagnosis and treatment of vitamin B12 deficiency: an update. Haematologica.

[3] Headstrom PD, Rulyak SJ, Lee SD (2008). Prevalence of and risk factors for vitamin B(12) deficiency in patients with Crohn’s disease. Inflamm Bowel Dis.

[4] Stabler Sally P (2013). Vitamin B12 deficiency. N Engl J Med.

[5] Lindenbaum J, Healton EB, Savage DG (1988). Neuropsychiatric disorders caused by cobalamin deficiency in the absence of anemia or macrocytosis. N Engl J Med.

[6] Healton EB, Savage DG, Brust JC, Garrett TJ, Lindenbaum J (1991). Neurologic aspects of cobalamin deficiency. Medicine.

[7] Snow FC (1999). Laboratory diagnosis of vitamin B12 and folate deficiency. Arch Intern Med.

[8] Green R, Kara N, Cocks H (2011). Vitamin B, deficiency: an unusual cause of vocal fold palsy. J Laryngol Otol.

[9] Wong CL, Van Spall HG, Hassan KA, Coret-Simon J, Sahlas DJ, Shumak SL (2008). A young man with deep vein thrombosis, hyperhomocysteinemia and cobalamin deficiency. Can Med Assoc J.

[10] Molloy A, Cawley N, Ali E, Connolly S, Tubridy N, Hutchinson M (2009). A pernicious leucoencephalopathy. Ir Med J.

[11] Celik M, Barkut IK, Oncel C, Forta H (2003). Involuntary movements associated with vitamin B12 deficiency. Parkinsonism Relat Disord..

[12] Srikanth SG, Jayakumar PN, Vasudev MK, Taly AB, Chandrashekar HS (2002). MRI in subacute combined degeneration of spinal cord: a case report and review of literature. Neurol India.

[13] Goebels N, Soyka M (2000). Dementia associated with vitamin B12 deficiency: presentation of two cases and review of the literature. J Neuropsychiatry Clin Neurosci.

[14] Berry N, Sagar R, Tripathi BM (2003). Catatonia and other psychiatric symptoms with vitamin B12 deficiency. Acta Psychiatr Scand.

[15] Sahoo MK, Avasthi A, Singh P (2011). Negative symptoms presenting as neuropsychiatric manifestation of vitamin B12 deficiency. Indian J Psychiatry..

[16] Milanlioglu A (2011). Vitamin B12 deficiency and depression. J Clin Exp Investig.

[17] Wilkinson Iain, Lennox Graham (2005). Essential Neurology.

[18] Lindebaum J, Nath BJ (1980). Megaloblastic anaemia and neutrophil hypersegmentation. Br J Haematol.

[19] Pawlak R, James PS, Raj S, Cullum-Dugan D, Lucas D (2013). Understanding vitamin B12. Am J Lifestyle Med..

[20] Thompson WG, Cassino C, Babitz L (1989). Hypersegmented neutrophils and vitamin B12 deficiency. Acta Haematol.

[21] Lindebaum J, Savage DG, Stabler BP (1990). Diagnosis of cobalamin deficiency;relative sensitivities of serum cobalamin, methylmalonic acid and total homocystein concentration. Am J Hematol.

[22] Berlin H, Berlin R, Brante G, Pilbrant A (1978). Vitamin B12 body stores during oral and parenteral treatment of pernicious anaemia. Acta. Med. Scand..

[23] Robert C, Brown DL (2003). Vitamin B12 deficiency. Am Fam Phy.

[24] Lee GR, Lee GR (1993). Megaloblastic and non megaloblastic macrocytic anaemias. Clinical Hematology.

